# Incidental Finding of Ectopic Adrenal Cortical Tissue During Orchidectomy for Intra-abdominal Cryptorchidism in a Nine-Year-Old Boy

**DOI:** 10.7759/cureus.114842

**Published:** 2026-08-20

**Authors:** Samia Benmali, Anass Haloui, Nassira Karich, Amal Bennani

**Affiliations:** 1 Department of Pathology, Faculty of Medicine, Mohammed VI International University Hospital, Mohammed First University, Oujda, MAR; 2 Department of Pathology, Mohammed VI International University Hospital, Faculty of Medicine and Pharmacy of Oujda, Mohammed First University, Oujda, MAR

**Keywords:** ectopic adrenal tissue, histopathology, incidental finding, intra-abdominal cryptorchidism, orchidectomy, pediatric

## Abstract

Ectopic adrenal tissue is a rare developmental anomaly resulting from aberrant migration of primordial adrenal cells during embryogenesis and is usually asymptomatic, being most often detected incidentally during surgical procedures performed for other conditions. We report the case of a nine-year-old boy who underwent laparoscopic exploration for a non-palpable left testis. Due to the high intra-abdominal position and marked atrophy of the testis, an orchidectomy was performed. Gross examination revealed no significant abnormalities. Histological analysis showed atrophic seminiferous tubules along with a well-circumscribed focus composed of non-atypical adrenal cortical cells without a medullary component. Immunohistochemical staining was positive for Melan-A and inhibin, while calretinin showed only weak positivity, strongly supporting adrenal cortical differentiation and arguing against Leydig cell hyperplasia. Staining for neuroendocrine markers (chromogranin and neuron-specific enolase (NSE)) was negative, confirming the absence of adrenal medullary tissue. The postoperative course was uneventful, and routine follow-up was scheduled. This rare incidental finding highlights the importance of systematic histopathological examination of orchidectomy specimens. Although typically benign, the identification of ectopic adrenal tissue is essential for understanding developmental anomalies and avoiding potential diagnostic pitfalls.

## Introduction

Cryptorchidism, defined as failure of testicular descent into the scrotum, is among the most common congenital anomalies in boys [1]. Surgical management, including orchidopexy or orchidectomy, may be required for intra-abdominal or severely atrophic testes [2].

Ectopic adrenal tissue refers to the presence of adrenal cortical cells located outside the normal adrenal gland. It results from aberrant embryologic migration or sequestration of fragments of the developing adrenal cortex, most commonly along the path of gonadal descent [3]. While considered a rare developmental anomaly in the general population, ectopic adrenal rests are a well-recognized entity in pediatric surgery. Literature reports indicate an overall incidence ranging from 1% to 9.3% in children undergoing groin surgical explorations [4]. More specifically, during explorations for cryptorchidism, the incidence of finding ectopic adrenocortical tissue is reported to be 3.3% in boys aged 0-7 years, and 3.2% in boys aged 8-15 years [5].

The presence of ectopic adrenal cortical tissue in a testicular specimen presents a significant diagnostic challenge for pathologists. Ectopic adrenal cortical tissue can perfectly mimic primary testicular lesions, most notably Leydig cell hyperplasia or Leydig cell tumors, which are frequently encountered in the setting of cryptorchidism and testicular atrophy [6].

This distinction is particularly difficult because both entities share strikingly similar morphological features - such as nests of polygonal cells with abundant lipid-rich vacuolated cytoplasm - as well as a common steroidogenic immunohistochemical profile (e.g., Melan-A and inhibin positivity) [7]. Consequently, relying solely on routine hematoxylin and eosin (H&E) staining and basic immunohistochemical markers can easily lead to misdiagnosis.

Accurate identification requires careful architectural evaluation and the use of immunohistochemical markers, including calretinin, which, when used in combination with other adrenocortical markers (e.g., Melan-A and inhibin), helps distinguish adrenocortical rests from Leydig cell proliferations [8]. This distinction is of major clinical importance because it may help avoid unnecessary radical surgical interventions and may prompt a necessary clinical workup for underlying systemic endocrine disorders, such as congenital adrenal hyperplasia (CAH) [9]. We report an incidental finding of ectopic adrenal cortical tissue identified in an orchidectomy specimen from a nine-year-old boy with intra-abdominal cryptorchidism and briefly discuss the relevant embryological and pathological aspects.

## Case presentation

A nine-year-old boy was referred for evaluation of a non-palpable left testis. Ultrasonography of the groin and abdomen confirmed the absence of the left testis from the scrotum and inguinal canal, while the right testis was normally positioned and of normal size. Laparoscopic exploration subsequently revealed a markedly atrophic left intra-abdominal testis attached to a very short spermatic cord. Because the testis was located intra-abdominally and could not be adequately mobilized for orchidopexy, orchidectomy was performed. The available operative report did not provide further details regarding the precise intra-abdominal location of the testis, the appearance or location of the adrenal glands, or the presence of other abnormalities.

**Figure 1:**
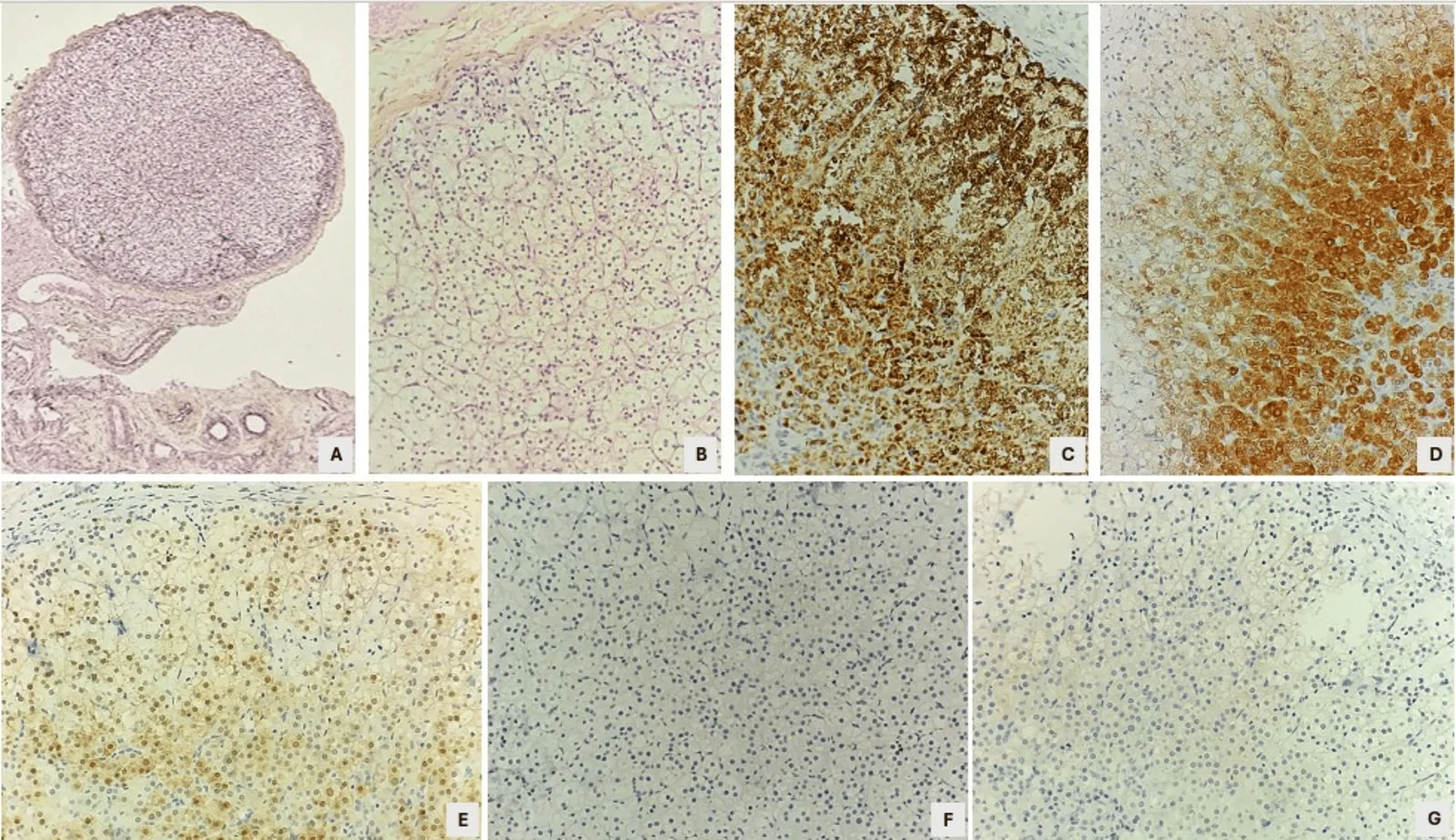
Histological and immunohistochemical features of ectopic adrenal cortical tissue (A) Low-power view showing a well-circumscribed focus of ectopic adrenal cortical tissue adjacent to atrophic testicular structures (HES stain, ×4). (B) Higher magnification view demonstrating nests of non-atypical polygonal cells with abundant clean-to-eosinophilic vacuolated cytoplasm containing lipid vacuoles. Note the classic organoid zonal architecture with cord-like structures resembling the zona fasciculata of the adrenal cortex and the absence of Reinke crystals (HES stain, ×20). (C) Immunohistochemical staining showing diffuse positivity for Melan-A, supporting steroidogenic differentiation (×20). (D) Immunohistochemical staining showing diffuse positivity for inhibin, supporting steroidogenic differentiation (×20). (E) Calretinin staining showing only weak positivity, supporting adrenal cortical differentiation (×20). (F) Negative chromogranin staining, confirming the absence of an adrenal medullary component (×20). (G) Negative neuron-specific enolase (NSE) staining, confirming the absence of an adrenal medullary component (×20).

Gross examination of the specimen revealed no significant abnormalities. Histological examination showed atrophic seminiferous tubules associated with a well-circumscribed focus composed of non-atypical polygonal cells. Importantly, the lesion displayed a classic organoid zonal architecture, with cord-like structures resembling the zona fasciculata of the adrenal cortex, helping to morphologically distinguish it from Leydig cell hyperplasia (Figures 1A-1B). The cells exhibited abundant clear-to-eosinophilic vacuolated cytoplasm containing lipid vacuoles. Notably, no Reinke crystals were identified. Furthermore, there was no evidence of cytological atypia, mitotic activity, or infiltrative growth.

Immunohistochemical analysis demonstrated diffuse positive Melan-A and inhibin expression (Figures 1C-1D). Calretinin staining showed only weak positivity (Figure 1E). Staining for the neuroendocrine markers chromogranin and neuron-specific enolase (NSE) was negative (Figures 1F-1G).

The postoperative course was uneventful, and the patient was discharged with routine follow-up.

## Discussion

Ectopic adrenal tissue is a rare developmental anomaly resulting from aberrant migration or sequestration of adrenal cortical cells during embryogenesis [3]. During fetal development, the adrenal cortex originates from the coelomic mesothelium near the urogenital ridge, in close proximity to the developing gonads. As the testes descend, fragments of adrenal cortical tissue may become displaced along the path of gonadal migration, explaining the frequent localization of ectopic adrenal rests within the spermatic cord, paratesticular region, or testis [7].

Ectopic adrenal tissue is most commonly identified in pediatric patients and is usually discovered incidentally during surgical procedures performed for cryptorchidism or inguinal hernia repair [4]. Most lesions are clinically silent and small in size, which explains why they are often overlooked during gross examination [7]. In the present case, the ectopic adrenal tissue was detected only after histopathological examination of the orchidectomy specimen, emphasizing the importance of systematic microscopic evaluation.

Histologically, ectopic adrenal cortical tissue is composed of well-circumscribed nests or cords of polygonal cells with abundant clear-to-eosinophilic vacuolated cytoplasm related to intracellular lipid content [3]. Cytological atypia, mitotic activity, and infiltrative growth are typically absent. These benign histological findings are also consistent with the classical pathological descriptions of adrenal cortical tissue [10].

The main diagnostic challenge in this context is differentiating ectopic adrenal cortical tissue from Leydig cell hyperplasia, Leydig cell tumors, and testicular adrenal rest tumors (TARTs), all of which may exhibit overlapping steroidogenic morphology and immunophenotypic features [11,12]. Leydig cell hyperplasia is a direct and very frequent consequence of testicular atrophy and cryptorchidism due to tubular retraction [6,13]. Morphologically, both entities share a similar appearance of polygonal cells with abundant clear-to-eosinophilic vacuolated cytoplasm. Although the absence of Reinke crystals in our case is consistent with adrenal tissue, these pathognomonic inclusions are only found in a minority of Leydig cell lesions, limiting their negative predictive value [12]. Therefore, identifying specific architectural features, such as the organoid zonal architecture seen in our case, is crucial.

Furthermore, a rigorous immunohistochemical panel is mandatory. While both tissues share a common steroidogenic immunophenotype (e.g., Melan-A and inhibin positive), they differ significantly in other markers. As noted in the literature, calretinin shows strong diffuse cytoplasmic and nuclear positivity in Leydig cells, whereas benign adrenal cortical rests are generally negative or only weakly positive [12]. In our case, the weak calretinin staining, together with the characteristic organoid architecture and the overall immunohistochemical profile, strongly supported the diagnosis of ectopic adrenal cortical tissue over Leydig cell hyperplasia. Additionally, the negativity of chromogranin and neuron-specific enolase confirmed the absence of an adrenal medullary component in the ectopic tissue [7]. The diagnosis should rely on the integration of the clinical context, histomorphological findings, characteristic architectural features, and immunohistochemical results rather than on any single marker.

Beyond Leydig cell lesions, the differential diagnosis should also include TARTs, particularly in pediatric patients, as these lesions may closely resemble other steroidogenic testicular lesions and have important clinical implications. This distinction has been emphasized in standard references on pediatric testicular pathology [11]. While both share an identical adrenal cortical origin and immunophenotype, TARTs are typically bilateral, located within the rete testis, and strongly associated with underlying systemic endocrine disorders, most notably CAH [9]. In contrast, ectopic adrenal rests, as seen in our patient, are usually unilateral, and most often purely incidental developmental anomalies [7].

Finally, in the setting of an intra-abdominal testis in a child, the presence of a specialized or monodermal mature teratoma must be explicitly considered. While immunohistochemical markers, such as SALL4 or AFP, are highly valuable to formally exclude a germ cell origin [14], they were unavailable in our laboratory. Therefore, the exclusion of a teratoma relied on extensive macroscopic sampling and rigorous morphological evaluation, which failed to reveal any other endodermal, ectodermal, or mesodermal components typically seen in teratomas [15].

Ultimately, the accurate recognition of ectopic adrenal tissue is essential to avoid misdiagnosing a benign developmental anomaly as a primary neoplastic process [12]. Such a misdiagnosis could lead to unwarranted radical surgeries, unnecessary oncological staging, and significant parental anxiety. Management primarily depends on the associated testicular abnormality [4]. In the present case, orchidectomy was performed because of the high intra-abdominal position, severe testicular atrophy, and impossibility of adequate mobilization due to the short spermatic cord. Although ectopic adrenal tissue is generally benign, rare cases of hyperplasia and neoplastic transformation have been reported [7], particularly in patients with endocrine disorders such as CAH [9].

## Conclusions

Ectopic adrenal cortical tissue is a rare but benign developmental anomaly that may be encountered incidentally in pediatric patients undergoing surgery for cryptorchidism. This case highlights the critical importance of systematic histopathological examination and the necessity of an extended immunohistochemical panel. Accurate diagnosis requires integration of histomorphological findings with an appropriate immunohistochemical panel, as ectopic adrenal tissue can closely mimic Leydig cell hyperplasia. Awareness of this entity, its specific architectural features, and the use of discriminatory markers like calretinin are essential to avoid diagnostic pitfalls, prevent misdiagnosis of primary testicular lesions, and guide appropriate clinical follow-up.
